# Prevalence of methicillin-resistant *Staphylococcus aureus* (MRSA) in respiratory cultures and diagnostic performance of the MRSA nasal polymerase chain reaction (PCR) in patients hospitalized with coronavirus disease 2019 (COVID-19) pneumonia

**DOI:** 10.1017/ice.2020.440

**Published:** 2020-08-26

**Authors:** Chitra D. Punjabi, Theresa Madaline, Inessa Gendlina, Victor Chen, Priya Nori, Liise-anne Pirofski

**Affiliations:** 1 Division of Infectious Diseases, Department of Medicine, Albert Einstein College of Medicine and Montefiore Medical Center, Bronx, New York; 2 Department of Pharmacy, Montefiore Medical Center, Bronx, New York

*To the Editor*—The need for studies on coronavirus disease 2019 (COVID-19) superinfections that can inform rational antimicrobial treatment and stewardship strategies has been recognized.^1^ In a recent review from our institution,^2^ we found that up to 71% of patients admitted with COVID-19 received antibiotics. Anti–methicillin-resistant *Staphylococcus aureus* (anti-MRSA) agents, particularly vancomycin, are important stewardship targets, and they are included in the 2019 World Health Organization (WHO) Watch List of Antibiotics.

Recently, guidance was published on the treatment of possible concomitant community-acquired bacterial pneumonia (CAP) for patients admitted with COVID-19.^3^ The authors recommend selective use of anti-MRSA therapy, as for any other patient with CAP, and the utility of the MRSA nares PCR is not addressed. However, the empiric use of these agents for admitted COVID-19 patients remains prevalent, driven by several factors. For one, staphylococcal superinfection is a common complication of other viral pneumonias, such as influenza,^4^ and the 2018 Infectious Diseases Society of America (IDSA) guidelines on influenza treatment state that “agents with activity against MRSA should be included in the empiric regimen for critically ill patients.”^5^ Additionally, the real-world treatment of patients with COVID-19 is complicated by recurrent fevers, fluctuating oxygen requirements, prolonged hospitalization and/or ventilation, blurring the line between community-acquired versus hospital-acquired pneumonia. Therefore, we sought to determine the prevalence of MRSA in respiratory cultures of patients admitted with COVID-19, and we evaluated the diagnostic performance of the MRSA nares PCR test, a valuable stewardship tool for ruling out MRSA pneumonia in low-prevalence settings,^6^ in this cohort.

## Methods

We conducted a retrospective, cohort study including adult patients admitted with COVID-19 (SARS-CoV-2 PCR test positive) between March 13, 2020, and May 17, 2020, across all campuses of Montefiore Medical Center (MMC), an academic center in the Bronx, New York. To determine the prevalence of MRSA in respiratory cultures, we included all patients with respiratory cultures obtained within 3, 7, 14, or 28 days of admission, and we calculated prevalence for each of these time points. To determine the diagnostic performance of the MRSA nares PCR, we included all COVID-19 patients who had MRSA nares PCR performed and a respiratory culture obtained within 5 days of the PCR test, at any point during their hospitalization. Data were obtained through Clinical Looking Glass, a computerized healthcare surveillance software at MMC linked to the electronic health record.

## Results

During the study period, 4,221 adult patients were admitted with COVID-19; only 472 patients (11.1%) had a respiratory culture. Patients with respiratory cultures were more severely ill than the patients without respiratory cultures, 78% of patients were intubated versus only 12% of those without respiratory cultures. Additionally, patients with respiratory cultures had longer lengths of stay (median, 19.5 days vs 6 days) and higher mortality (56% vs 21.5%) versus those who had no respiratory culture. Overall, 904 (21.4%) received empiric vancomycin within 48 hours of admission, and this was more commonly seen in the group that had respiratory cultures obtained (33.4%). Patient characteristics are summarized in Supplementary Table 1 (online).

Among the 4,221 patients in the entire cohort, 158 patients had respiratory cultures obtained by day 3 of hospitalization, 285 by day 7, 405 by day 14, and 472 by day 28. The prevalence of MRSA in respiratory cultures ranged from a low of 0.6% on day 3, to 5.7% on day 28, cumulatively (Table 1).

**Table 1.** Prevalence of Methicillin-Resistant *Staphylococcus aureus* (MRSA) in Respiratory Cultures at Different Time Points of Hospital Stay

Days from Admission	Day 3	Day 7	Day 14	Day 28
Total patients with respiratory cultures obtained, no.	158	285	405	472
Patients with MRSA in respiratory cultures, no	1	7	18	27
Prevalence, %	0.6	2.4	4.4	5.7

Also, 369 MRSA nares PCR tests were performed among the patients in the cohort; of these patients, 122 had corresponding respiratory cultures within 5 days of the PCR test. Of these 122 MRSA PCR tests, 12 were positive, of which 2 patients had a corresponding positive respiratory culture for MRSA. Of the 110 negative nasal MRSA PCR tests, none of the corresponding respiratory cultures had MRSA isolated, yielding a negative predictive value (NPV) of 100% (Supplementary Table 2 online).

## Discussion

Patients requiring hospitalization for COVID-19 are often admitted with a severe pneumonia and critical illness. Given the severity of illness and a lack of data on superinfections, MRSA is often a clinical concern, and we noted frequent empiric use of vancomycin, especially in those who were more severely ill.

Respiratory cultures were not frequently obtained, reflecting the concern that suctioning or sputum induction would cause aerosolization. Those who got respiratory cultures, however, tended to be more severely ill, the very group in which a MRSA coinfection would be most concerning. Even in this more severely ill group, MRSA was not commonly identified as a respiratory copathogen, especially early in the course of admission. The prevalence increased with prolonged hospital stay, implying that it is more likely to be a hospital-acquired or ventilator-associated complication than a community-acquired coinfection. Nevertheless, rates still remained low further into admission. Our findings suggest that continued empiric usage of vancomycin for pneumonia in patients with COVID-19 is likely not warranted. Clinicians should remain guided by local epidemiologic data; notably, however, the Bronx has had the highest rates of MRSA infections in New York City.^7^ Additionally, our findings are in keeping with decreasing rates of MRSA infections across the United States in recent years.^8,9^

Given the low prevalence, we found excellent diagnostic performance of the MRSA nares PCR test, with 100% negative predictive value, confirming that the MRSA nares PCR test remains an important stewardship tool to guide discontinuation of anti-MRSA antibiotics, if started empirically for pneumonia in patients with COVID-19.

## Supplementary material

For supplementary material accompanying this paper visit https://doi.org/10.1017/ice.2020.440. click here to view supplementary material
