# Supplementary material for: Prevalence of methicillin-resistant Staphylococcus aureus (MRSA) in respiratory cultures and diagnostic performance of the MRSA nasal polymerase chain reaction (PCR) in patients hospitalized with coronavirus disease 2019 (COVID-19) pneumonia
Source: Infect Control Hosp Epidemiol. 2020 Aug 26:1–2. doi: 10.1017/ice.2020.440 (PMC7588710; doi:10.1017/ice.2020.440)
Supplement: Supplementary file 1 [file S0899823X20004407sup001.docx]

Supplementary Table 1: Performance of the MRSA Nares PCR test in COVID-19 patients

|  | **MRSA in respiratory cultures?** | | Sensitivity | 100% |
| --- | --- | --- | --- | --- |
| MRSA PCR Test | No | Yes | Specificity | 91.6% |
| Not Detected | 110 | 0 | PPV | 16.7% |
| Detected | 10 | 2 | NPV | 100% |
